# Cell-Based Therapeutic Approaches for Cystic Fibrosis

**DOI:** 10.3390/ijms21155219

**Published:** 2020-07-23

**Authors:** Pascal Duchesneau, Thomas K. Waddell, Golnaz Karoubi

**Affiliations:** 1 Latner Thoracic Surgery Research Laboratories, Toronto General Hospital Research Institute, University Health Network, 101 College St., Toronto, ON M5G 1L7, Canada; pascal.duchesneau@uhnresearch.ca (P.D.); Tom.Waddell@uhn.ca (T.K.W.); 2 Institute for Biomaterials and Biomedical Engineering, University of Toronto, 164 College Street, Toronto, ON M5S 3G9, Canada; 3 Institute of Medical Science, University of Toronto, 1 King’s College Circle, Toronto, ON M5S 1A8, Canada; 4 Department of Laboratory Medicine and Pathobiology, University of Toronto, 1 King’s College Circle, Toronto, ON M5S 1A8, Canada; 5 Department of Mechanical and Industrial Engineering, University of Toronto, 5 King’s College Road, Toronto, ON M5S 3G8, Canada

**Keywords:** cystic fibrosis, cell therapy, lung, airway, autologous, allogeneic, induced pluripotent stem cells, mesenchymal stromal cells

## Abstract

Cystic Fibrosis (CF) is a chronic autosomal recessive disease caused by defects in the cystic fibrosis transmembrane conductance regulator gene (*CFTR*). Cystic Fibrosis affects multiple organs but progressive remodeling of the airways, mucus accumulation, and chronic inflammation in the lung, result in lung disease as the major cause of morbidity and mortality. While advances in management of CF symptoms have increased the life expectancy of this devastating disease, and there is tremendous excitement about the potential of new agents targeting the *CFTR* molecule itself, there is still no curative treatment. With the recent advances in the identification of endogenous airway progenitor cells and in directed differentiation of pluripotent cell sources, cell-based therapeutic approaches for CF have become a plausible treatment method with the potential to ultimately cure the disease. In this review, we highlight the current state of cell therapy in the CF field focusing on the relevant autologous and allogeneic cell populations under investigation and the challenges associated with their use. In addition, we present advances in induced pluripotent stem (iPS) cell approaches and emerging new genetic engineering methods, which have the capacity to overcome the current limitations hindering cell therapy approaches.

## 1. Introduction

Lung diseases are major challenges to the health care sector, and the second leading cause of death in our society. For patients with end-stage lung disease, direct costs are high [1] and lung transplantation has become both a cost-effective treatment approach [2] and often the only life-saving option, despite associated risks of mortality and morbidity due to graft rejection and infection. Cystic Fibrosis (CF), caused by mutations in the gene encoding CF transmembrane conductance regulator (*CFTR*), is the most common life-limiting, autosomal recessive monogenic disease in Caucasian populations [3]. While the loss of *CFTR* function affects multiple organs including the lungs, pancreas, liver, and intestine, progressive lung disease and respiratory failure are the major cause of morbidity and mortality for most patients [4]. Recent advances in pharmacological agents such as *CFTR* correctors and potentiators (reviewed in Gentzsch et al. (2018) and Burgener et al. (2018)) [5,6] and other medical advancements, including lung transplantation, have extended the mean survival of CF patients. However, patients are still faced with reduced quality of life, severe pulmonary complications, and the high costs associated with the lifelong intake of drugs. These limitations and the difficulties of the transplant option continue to drive the search for a more fundamental “cure”.

**Figure 1:**
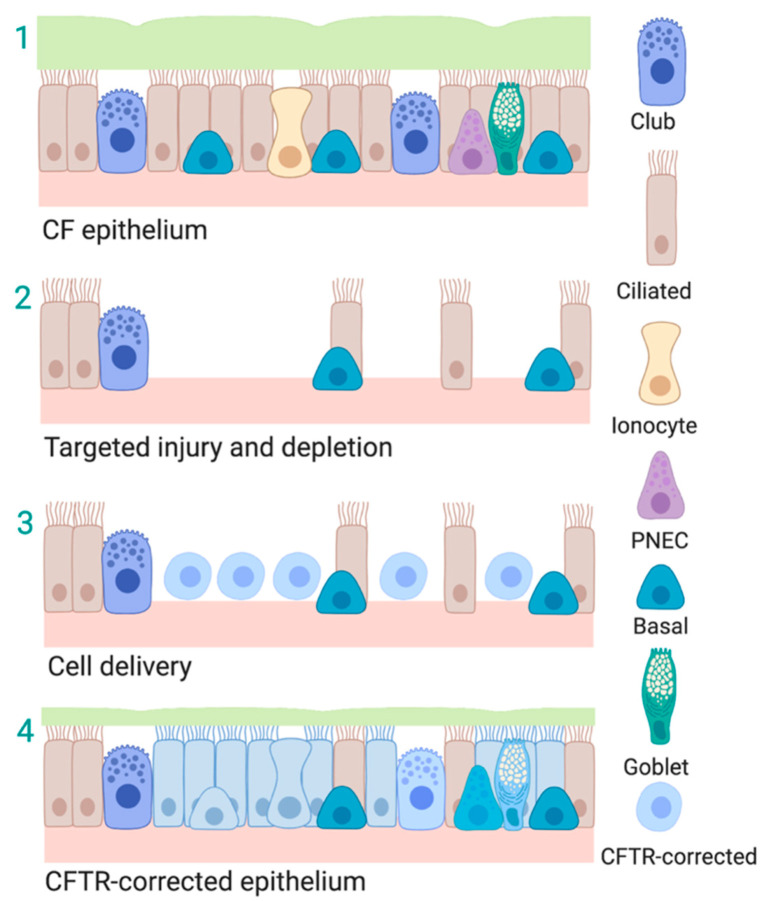
Schematic of cell-based replacement therapy for Cystic Fibrosis. Cell-replacement therapy in which defective airway epithelium (1) is ablated via injury to the airways thereby creating a niche for engraftment (2). Corrected cells are then delivered into the airways, localize and engraft in the exposed niche (3) and restore functional epithelium (4). Figure created with BioRender.com.

The most important problem in CF is the defective function of *CFTR* protein in epithelial cells of the smallest airways. Rather than fixing *CFTR* in the airway cells, cell replacement therapy would replace them. Mechanistically, the approach could reduce disease impact either via replacement of the defective chloride transport, seen with mutation of the *CFTR* gene, or lessen the impact of secondary mediators of inflammation. Intact but genetically defective epithelium in the CF airway would be selectively targeted for removal, allowing replacement with progenitor cells with corrected *CFTR* (Figure 1). This strategy is analogous to hematopoietic stem cell transplantation following cytotoxic chemotherapy, to create “space” in the bone marrow niche. The lung is an ideal organ system for cell-therapy approaches, since minimally invasive access by bronchoscopy, allows us to deliver cells and monitor their persistence directly and potentially their efficacy. Encouragingly, Johnson et al. [7] and others [8] showed that correction of *CFTR* in only a fraction of cells may be sufficient to restore electrophysiological function and permanently improve clinical outcome. Thus, the concept of “cell replacement therapy” for CF continues to be a scientifically valid and clinically relevant goal. 

In this review, we will outline the current state of cell-based therapeutic approaches in the CF field. We will first review the existing pre-clinical animal models of CF and their utility in cell therapy. We will highlight the different cell sources used as vectors in these models and the challenges associated with their use. We will then discuss emerging new ‘designer cells’ from pluripotent sources, molecularly engineered to address some of the current limitations. 

## 2. Etiology and Pathophysiology of CF

Cystic Fibrosis is an autosomal recessive disease caused by mutations in the *CFTR* gene involved in chloride and bicarbonate transport. CF affects multiple organs such as intestine, pancreas, liver and gallbladder but lung disease is the major cause of morbidity and mortality as a result of mucus accumulation, chronic inflammation, and persistent bacterial infection [9,10,11]. Although CF is caused by mutations in a single gene, over 2000 genetic variants have been identified [12]. Those mutations are classified into six groups according to the synthesis, trafficking, and function of *CFTR* which include (1) no synthesis, (2) defective processing, (3) defective gating, (4) low conductance, (5) low synthesis, and (6) increased turnover. The most common mutation, a deletion of phenylalanine 508 (ΔF508), accounts for approximately 85% of CF cases and can be classified in multiple groups adding to the complexity. Moreover, patients with the same genetic variation may exhibit different clinical phenotypes attributed to environmental factors and modifier genes [13].

With respect to the epithelium, progressive remodeling of the airways ultimately results in structural damage and impaired lung function and it is unclear whether these changes are related to and initiated by infection/inflammation or are a result of *CFTR* dysfunction [14,15]. Hyperplasia of goblet and basal cells, squamous metaplasia, increased epithelial height, cell shedding, loss of ciliated epithelial cells, and a disorganization of tight junctions and compound cilia have been reported. In addition, extensive structural changes of the small airway epithelia have also been observed, including epithelial shedding and altered barrier integrity (reviewed in De Rose (2018)) [16]. 

It is also not clear which *CFTR*-expressing cells are responsible for CF disease thus making targeted therapeutic approaches challenging. Some studies have suggested that *CFTR*-dependent submucosal gland secretions have an important role in airway innate immunity [17,18]. More recently, rare *FoxI1^+^* pulmonary ionocytes which are rich in *CFTR* and account for less than 1% of epithelial cells have been described [19,20]. Ionocytes are believed to have an important role in fluid regulation of airway surfaces and appear to be replenished by basal cells. For significant advancements in cell-based therapies for CF, a better understanding of the most important cell populations to target and ways to remove them will be needed. 

## 3. Animal Models of CF 

Animal models are an essential tool to study the pathophysiology of CF and for the development of treatment methodologies. Following cloning of the CF gene in 1989, the first animal model was introduced in mice 3 years later [21,22]. These *CFTR*-deficient mice quickly developed lethal intestinal obstruction and needed to be fed a liquid diet. To facilitate husbandry and improve survival for lung studies, a gut-corrected model was introduced 2 years later [23]. Despite showing some abnormalities in the lung following bacterial challenge, *CFTR*-deficient mice did not recapitulate the characteristic lung pathophysiology observed in human patients. This was thought to be the result of a redundant chloride transport channel in murine lungs. Since then, multiple animal models, including conditional *CFTR* knockout models, have been developed in mice, pigs, zebrafish, rats, rabbits, and sheep [24,25,26,27,28,29].

The pig model showed early signs of airway inflammation, airway remodeling, mucus accumulation, and infection with multiple bacterial species and is 100% lethal without ileostomy performed after birth [9,30]. Thus, a second generation *CFTR* pig was developed with gut-correction [31]. Similarly, to the CF pigs, *CFTR*-deficient ferrets also show evidence of lung infections in early life necessitating antibiotic treatment prior to weaning and therefore a gut-corrected second generation was developed [32,33]. Sheep and rabbit CF models are relatively new models and have not yet been fully characterized. Since the sheep has similar lung anatomy to humans it could be a promising model while the rabbit could be useful for fast reproduction and availability of multiple antibodies. However, early results in newborn *CFTR*^−/−^ sheep showed lethal pancreatic fibrosis, intestinal obstruction, and absence of the vas deferens suggesting that a gut-corrected second generation model may be required [27]. 

Currently, no single animal model recapitulates all aspects of human CF disease and the majority of cell-based therapeutic studies in the lung have been performed in mice. Larger animal models, such as pig and ferret, are resource-intensive and challenging to raise but may be the most useful preclinical models to study cell-based therapies in a chronic lung infection environment. To our knowledge, no cell therapy has been performed in these models with only few studies focusing on gene therapy. As the field moves towards developing clinically relevant cell-based approaches, large animal models of CF will become useful in assessing cell-based therapies. These models will need to both better recapitulate the human disease and allow for testing of conditioning protocols to remove target epithelium and allow for retention and long-term engraftment of transplanted cells.

## 4. Overview of Cell Types Used in Cell-Based Therapeutic Approaches for the Lung

The mature lung comprises at least 40 morphologically and functionally distinct cell populations including epithelial, inflammatory, stromal, and endothelial cells [34,35]. Epithelial cells include those forming alveolar units, the Type I and II alveolar epithelial cells, and those lining proximal airways, including ciliated, mucous, Club, basal, and pulmonary neuroendocrine cells (PNEC). Identifying epithelial stem cells in the lung has been difficult due to low cell turnover, but by assessing cell proliferation and using ‘lineage tagging’ techniques [34,35,36,37,38,39], various niches have been identified that contain stem or progenitor cells. Despite the marked progress, the field remains limited in its ability to produce therapeutically applicable cell numbers derived from endogenous stem cells hindering their use in cell-based applications.

The majority of lung regenerative medicine studies have focused on the use of exogenous cell types, predominantly on the use of bone marrow cells (BMC) and in particular the adherent stromal population referred to as mesenchymal stem cells and/or mesenchymal stromal cells (MSC). Engraftment and the significant therapeutic effects of MSC has been shown following various human and experimental lung injuries [40,41,42,43,44,45,46,47,48,49,50]. Although in the majority of cases, therapeutic effects have been observed, there is still considerable debate about the fate of the cells [51,52,53,54], the true level of cell engraftment [42,43,44,45,51,52,53,54], and whether transplanted MSC can truly replace epithelial cells.

As the lung is a complex organ, pluripotent cells would serve as ideal therapeutic units for lung regeneration. There have been significant efforts put forth in generating lung epithelium using embryonic stem (ES) cells and induced pluripotent stem (iPS) cells [55,56,57,58,59,60,61,62,63,64], and recent progress in directed differentiation studies has indicated their potential for use as a cell source for treatment in lung injury models [58,60,65,66,67].

## 5. Mesenchymal Stromal Cells (MSC)

The bone marrow is the main source for hematopoietic stem cells, harbors endothelial progenitor cells as well as MSC, which represent 0.001–0.01% of the nucleated cells in the marrow [68]. Since their identification in the bone marrow, MSC have been isolated in a variety of other tissues including umbilical cord blood, placental, and adipose tissues [69,70,71]. Data suggesting that these cells can engraft and develop into cell types of other organs including the heart [72], brain [73], liver [74], pancreas [75], skin [76], and lung [77,78,79,80,81,82] has been presented. Most of these observations were based upon co-localization of tissue specific phenotypic markers with sex, transgenically, or dye-marked cells. These reports led to wide interest in BMC therapy for lung injury.

Many groups have since reported much lower lung engraftment of MSC leading to some skepticism in the efficiency of these cells to regenerate epithelium [79,83,84,85,86]. The difficulty in reproducibility of the reported engraftment levels and the possibility of cell fusion has added to the controversy surrounding MSC plasticity. This may have been due to differences in methodologies used, lack of proper controls, and artifacts [86]. Despite concerns regarding lack of standardized methodologies and wide range of variability in observed engraftment potential, the biological effects of BMC are less easily ignored. Therapeutic benefit has been shown following various human and experimental lung injuries such as lung transplantation [87], endotoxin-induced acute lung injury [79,88,89,90,91,92,93], asthma [94,95,96], bronchopulmonary dysplasia [97,98], emphysema [99,100], bleomycin-induced fibrosis [78,97,101,102,103,104,105,106], sepsis [45,107,108,109], and ventilator-induced injury [107,110].

Current thinking suggests that while BMC and MSC are not able to fully transdifferentiate, BMC may promote lung tissue repair through the ability to adopt the expression profiles and functional phenotypes of lung cells even if only temporarily, or via paracrine effects—the ability to secrete soluble growth factors, cytokines, and even organelles which can exert their influence on lung repair. Bioactive factors secreted by MSC are known to mediate immunomodulatory, anti-inflammatory, antioxidant, and anti-apoptotic effects. They also contribute to tissue regeneration, angiogenesis, and clearance of microorganisms. Indeed, the secretory and paracrine effects of MSC have continued to be extensively studied, and it is widely accepted in the MSC field that their secretome is predominantly responsible for the intercellular crosstalk between MSC and targeted cells [111,112,113].

Our lab has contributed to this field with identification of a novel subset of bone marrow cells with particular utility in both mice and human airways [40,41,114,115,116,117]. To increase the therapeutic utility of cell replacement therapy, we have undertaken a number of experiments to optimize cell retention within the lung including a recipient conditioning regimen of naphthalene and busulfan treatment prior to transplantation [41,117]. We have been able to improve BMC retention efficiency by a factor of 10-fold, even in CF *CFTR* knockout recipient animals. With these technical improvements in delivery regimen, we have achieved substantial increases in expression of *CFTR* mRNA, and detectable *CFTR* protein. Importantly, we found that treatment of *CFTR*^−/−^ mice with *CFTR*^+/+^ BMC improved bacterial clearance resulting in greater survival of the *CFTR*^−/−^ mice treated with BMC [41]. However, despite this progress, proving that the transferred *CFTR* is functional in vivo has been extremely difficult. 

In the context of CF, Wang et al., suggested that MSC were able to differentiate into airway epithelial cells when co-cultured with primary human airway epithelial cells under air–liquid-interface (ALI) conditions. Importantly, the authors found that *CFTR*-corrected MSC (transduced with a lentiviral vector bearing a wild type *CFTR* gene) from homozygous ΔF508 CF patients were able to contribute to apical Cl^-^ secretion in response to cAMP agonist stimulation [118]. In subsequent studies by the Conese group, human amniotic MSC were also felt to differentiate into airway epithelial cells when co-cultured with CF immortalized airway epithelial cells [119,120]. Carbone et al. showed that MSC sourced from human amniotic mesenchymal stromal cells co-cultured in ALI with CF immortalized airway epithelial cells were able restore some of the basic defects associated with CF. Specifically, co-cultures had more organized tight junctions with increased expression of occludin and *ZO-1* and decreased dextran permeability and resumed chloride transport. 

Bonfield and colleagues have shown in a lung infection and inflammation mouse model of CF (*Pseudomonas aeruginosa* and *Staphylococcus aureus)*, that human MSC decreased the bacterial burden and thereby enhanced the ability of the CF lung to resolve the infection potentially through changes in the in vivo production of the antimicrobial peptide LL-37. They also showed in vitro that supernatant from hMSC derived from both bone marrow and adipose tissue reduced bacterial growth of *Pseudomonas aeruginosa*, *Staphylococcus aureus*, and *Streptococcus pneumoniae* [121]. Further evaluation of the mechanistic action of hMSC showed that in vivo hMSC recruit macrophages known to be important in infection resolution and attenuation of the inflammatory response [122]. In vitro, they showed that hMSC decreased pro-inflammatory cytokine production in LPS-stimulated mouse bone marrow-derived macrophages or human peripheral blood mononuclear cells, and upregulate the expression of *PPARγ*, which is an important regulator of inflammation in chronic inflammatory diseases such as CF [122,123]. 

Studies have found that MSC secrete different types of extracellular vesicles (EV) believed to account for much of their therapeutic effects [124,125,126,127]. Additionally, more recent reports have explored the role of released EV from MSC as a potential therapeutic application for controlling inflammation in Cystic Fibrosis [128]. Zulueta and colleagues showed that treatment of IB3-1 CF cell line, (an in vitro human model of CF), with EV derived from human lung MSC under basal and inflammatory conditions (*TNFα* stimulation) downregulated transcription and protein expression of pro-inflammatory cytokines *IL-1β*, *IL-8*, *IL-6* and upregulated the mRNA expression of *PPARγ* (a transcription factor controlling anti-inflammatory and antioxidant mechanisms via *NF-kB* and *HO-1*). They also observed reduced *NF-kB* nuclear translocation and increased *HO-1* expression confirming the impairment of the downstream inflammation cascade [128]. 

Despite recent progress in small animal and in vitro studies, the utility of MSC secretome for treatment of CF remains to be determined and there remains skepticism that MSC will ever function as an equal replacement for respiratory epithelium. Additionally, although there have been numerous preclinical studies on using MSC for cell-based therapy in CF, the vast majority of these have been in small animal models. To determine whether MSC cell or secretome delivery is a viable option for treatment of CF, there needs to be further work in large animal models and in clinical trials. In fact, there are currently only two ongoing phase I clinical trials (NCT02866721 and NCT03058068) investigating MSC therapy in CF. The first, Safety and Tolerability Study of Allogeneic Mesenchymal Stem Cell Infusion in Adults With Cystic Fibrosis (CEASE-CF; NCT02866721), is a prospective, single-center, dose-escalation, open-label interventional study to evaluate the safety and tolerability of allogeneic hMSC in 15 clinically stable subjects with CF age ≥ 18 years. Results from this trial are yet to be published. The second trial, Human Mesenchymal Stem Cells Infusion in Patients With Cystic Fibrosis (HAPI; NCT03058068), focuses on the demonstration of the safety of MSC intravenously administered to 15 adult subjects with Cystic Fibrosis with a secondary objective to explore if MSC can improve the symptoms of cystic fibrosis, including lung function, the rate of pulmonary exacerbation, systemic and local inflammation, and symptom-related quality of life. It is important to note that since its initiation, the latter study has been withdrawn as the principal investigator is no longer at the institution.

## 6. Induced Progenitor-Like Cells (iPL)

**Figure 2:**
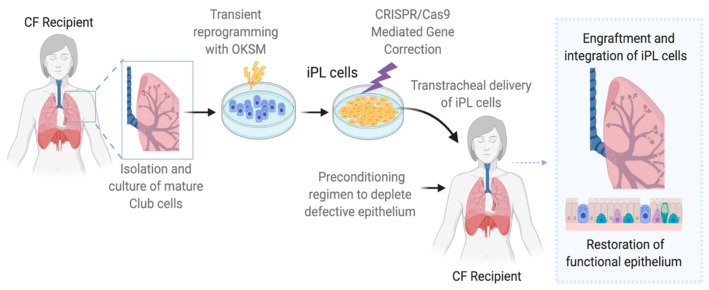
Schematic representation of the Induced progenitor-like cells (iPL) process and their utility in cell therapy for Cystic Fibrosis (CF). iPL are generated from mature club cells isolated from a CF patient via transient reprogramming with *Oct4*, *Sox2*, *Klf4*, and *c-Myc* (OKSM). Gene corrected iPL cells are then transplanted back to the preconditioned recipient airways where they will engraft and integrate into the airways and restore the epithelium. Figure created with BioRender.com.

Due to the limitations and skepticism surrounding MSC, as an alternate source of autologous cells, we have been working on a novel cell type which we have developed by careful dissection of events underlying reprogramming during iPS cell generation. Transient reprogramming with transcription factors *Oct4*, *Sox2*, *Klf4*, and *c-Myc* (OSKM) resulted in an intermediate product of the iPS cell process which we have termed “induced progenitor-like” (iPL) cells [129,130]. We noted that iPL cells are highly proliferative but retain epigenetic “memory” that allows return to their original identity upon withdrawal of reprogramming factors. Specifically, we isolated highly purified populations of Club cells from *R26-rtTA/Col1a1:tetO-4F2A* double transgenic mice enabling expression of OSKM. We used controlled, transient, exogenous activation of the transcription factors with doxycycline, which causes reprogramming towards iPS cells, but turns off the expression of the OSKM factors prior to reaching the commitment point leading to pluripotency. Throughout this process, we have generated and characterized iPL cell populations derived from the Club cells of the proximal airways [129], alveolar type II epithelial cells (AEC-II) of the distal lung parenchyma [130], and pulmonary endothelial cells (Unpublished data). We found that using Club cells as the starting population, these induced progenitor-like (iPL) cells can be expanded 30-fold yet differentiate normally to ciliated cells with functional expression of *CFTR* [129]. In vivo, we showed successful retention and incorporation of Club cell-derived iPL cells in airways of *CFTR*-deficient animals with injected iPL cells giving rise to both Club cells and ciliated cells [129]. A schematic depiction of the generation and utility of Club-cell derived iPL cells is shown in Figure 2. 

While we have made significant progress in generating and characterizing a viable source of cells that may be applicable in the clinic in the future, there are several challenges that this technology presents. These include the need for optimization of the partial reprogramming in the human system, as well as the required customization of the approach to any given somatic cell type. For CF patients, an additional step of gene correction will be required. Another limiting factor for autologous cell therapy as a whole, remains the time and effort in obtaining the required cells and the cost associated with the treatment. However, from an immunological perspective, autologous transplantation is ideal and thus it remains a valuable avenue to pursue.

## 7. Embryonic and Induced Pluripotent Stem Cells (iPS)

Unlike autologous cell sources, human pluripotent stem cells (hPSC) hold enormous promise to serve as the source of unlimited therapeutic cells used to treat injuries and degenerative diseases in future cell-based therapies. There have been significant efforts put forth in generating lung epithelium using pluripotent cell sources [55,56,57,58,59,60,61,62,64,65,66,131,132,133], with evidence for their potential use as a cell source for treatment of lung injury models [57,59,65,66,132,134,135,136,137,138]. Induced pluripotent cells are particularly advantageous to embryonic stem cells, as they are associated with less ethical controversy [139,140] and can be used as an autologous cell source.

**Figure 3:**
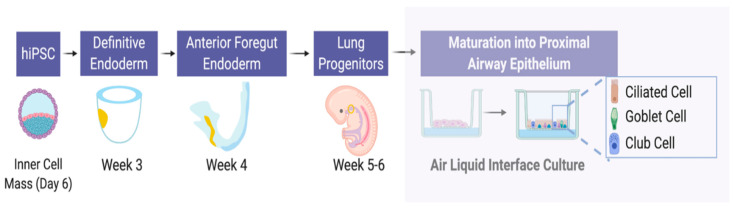
Derivation of proximal airway epithelial cells from induced pluripotent stem (iPS) cells. Schematic representation of the embryologically guided stepwise approach to generate mature proximal epithelial cells. The first requirement is the generation of definitive endoderm (equivalent to cells at approximately 3 weeks post conception) from iPS cells (equivalent to the cells found in the inner cell mass of the blastocyst at day 6). The second stage is differentiation to anterior foregut endoderm (equivalent to cells at approximately 4 weeks post conception). Cells are then differentiated via ventralization to *NKX2.1^+^* lung progenitors (equivalent to cells at approximately 5–6 weeks post conception). Proximal airway lineage and maturation is then induced using 3D culture conditions and air–liquid-interface (ALI) culture. Figure created with BioRender.com.

Most of the focus in the pluripotent stem cell field in relation to CF, has been on generation of proximal lung epithelial cells to develop patient-specific models of CF [58,60,63] and generation of multi-ciliated cells [61,62]. The protocols presented in these studies follow the basic premise of using the embryological development of the lung as a guide [141] but differ in their use of type, concentration, and time of exposure of chemical stimuli and growth factors. As such, most protocols guide pluripotent stem cells through the developmental stages of definitive endoderm, followed by anteriorization to foregut endoderm, subsequent ventralization to generate *NKX2.1^+^* putative lung progenitor cells, and finally maturation to proximal airway epithelium using air–liquid interface (ALI) culture in which cells are basally exposed by to media and apically to air (Figure 3). 

While early attempts using simpler 2D culture protocols showed successful differentiation into lung progenitor populations, mature epithelial cells were not obtained [60], with varying *CFTR* expression amongst iPS cells derived from CF patients [58]. Subsequent studies [61,131] using lower concentration of BMP4 required during the ventralization phase, also resulted in epithelium lacking multi-ciliated cells but did generate *CCSP*^+^ Club cells, *MUC5AC*^+^ goblet cells, and some functional *CFTR*^+^ cells [61]. This protocol was used to differentiate induced pluripotent stem cells (iPSC) from CF patients carrying a homozygous deletion of F508 in the *CFTR* gene (resulting in a defective processing of *CFTR* to the cell membrane) and corrected using clustered regularly interspaced short palindromic repeats (CRISPR)/CRISPR associated protein 9 (Cas9) genome editing to target corrective sequences to the endogenous *CFTR* genomic locus, using a completely excisable piggyBac transposase system [131]. The corrected iPS cells were subsequently differentiated to mature airway epithelial cells and analyzed for *CFTR* currents by whole cell patch clamp methods. In the CF iPS-derived epithelial cells, approximately 50% of the cells responded to stimulation via exposure to a cocktail of forskolin, genistein, and 3-isobutyl-1-methylxanthine (IBMX) [131]. 

More recent protocols have incorporated supplemental 3D culture in Matrigel as well as *NOTCH* inhibition, a maturation cue, during the ALI culture phase [62,63,64,142]. These protocols can achieve multi-ciliated cells with ciliary beating frequency similar to that of primary bronchial epithelial cells. These significant advances in directed differentiation protocols have enabled derivation and use of iPS-derived epithelium from CF patients. In their study, McCauley and colleagues generated airway spheroids from patient-specific CF lines (RC2 202 and RC2 204 lines both with homozygous ΔF508 *CFTR* mutations) as well as CF corrected iPS lines. They showed forskolin-responsive swelling in normal and not CF patient-derived spheroids and were able to rescue the defect in swelling via genome editing in the CF corrected iPS lines [63]. 

Although human iPS cells hold enormous promise as the source of unlimited therapeutic cells, there remain challenges in the purity and yield of the cells produced. For utility, standardized protocols are required which produce reproducible numbers of *CFTR* expressing cells potentially including the newly discovered ionocytes. In addition, prior to clinical application of iPS cells, significant hurdles with respect to their safety and acceptance by the immune system must be overcome.

## 8. Designer Pluripotent Cells 

The generation of designer pluripotent cells, in which iPS cells are molecularly and/or genetically engineered for altered or enhanced function offers the possibility to circumvent their existing limitations. Recent advances in genome editing technologies, particularly the CRISPR/Cas system, have facilitated the targeted integration of functional DNA elements into the human genome, thus, extending their research and therapeutic applications [143]. These include approaches to address both the safety as well as immunogenicity of pluripotent cell sources. The tumorigenicity of hPSC, mainly monitored as teratoma formation after in vivo injection, remains a major challenge that needs to be overcome for the application of hPSC in the clinic. Thus, a variety of approaches have been investigated with the aim to identify and eliminate undifferentiated hPSC including chemical ablation [144], targeting and removal of pluripotent-specific hPSC antigens with cytotoxic antibodies [145], genetic modification of tumor-driving genes [146], insertion of cytotoxic suicide genes [147], and the use of small molecule-based selective elimination [148]. More recently, Liang et al. (2018) described a cell-therapy ‘safety’ solution, termed the safe-cell (SF) suicide system. In this system, they achieve safety by transcriptionally linking a drug-inducible suicide system, Herpes Simplex Virus type 1 thymidine kinase (*HSV-TK*), to the *CDK1* cell division essential loci (essential for a cell to divide or to survive) using CRISPR/Cas9 genome editing; allowing complete control over proliferating cells. Therefore, if required, the dividing cells can be arrested or eliminated by treatment with the drug that induces the suicide system (in this case, ganciclovir (GCV) which activates the *HSV-TK* suicide system) [149].

In addition to iPS tumorigenicity and the risk of teratoma formation, immune rejection due to expression of human leukocyte antigens (HLA) remains a significant problem. These are encoded by a highly polymorphic set of genes and include *HLA* class I (*HLA-A*, *HLA-B*, and *HLA-C*) and class II (*HLA-DP*, *HLA-DM*, *HLA-DO*, *HLA-DQ*, and *HLA-DR*) molecules [150]. Approaches to generate iPSC stocks isolated from *HLA* homozygous donors are currently under exploration and it is believed to be possible to cover most *HLA* haplotypes [151], but the recruitment of *HLA* homozygous donors that serve an entire population is very difficult and require the generation of a large number of cell lines, extensive validation, and stringent regulatory processes [150]. Genetic manipulation of *HLA* gene expression for instance via knockout of β2-microglobulin (*β2m*, major component of *HLA* class I molecules) resulting in reduced expression of *HLA* genes has shown to be a promising approach [150,152,153]. Another approach has been to overexpress immunosuppressive genes [152,154]. For example, overexpression of immunosuppressive receptors cytotoxic T-lymphocyte-associated protein 4-immunoglobulin (*CTLA4-Ig*) and programmed death-ligand 1 (*PD-L1*) in human ESCs (hESCs) have been shown to prevent allogenic immune rejection [154]. Similarly, Deuse et al. demonstrated that human iPSCs lose their immunogenicity when *HLA* class I and II genes are inactivated via CRISPR/Cas9 disruption of *β2m* gene and *Ciita* (master regulator of HLA class II genes), and *CD47* is overexpressed [152].

While suicide gene approaches could eliminate tumor forming cells even after cell transplantation, their efficacy remains controversial. Another concern is the safety of genome editing. Our knowledge of human genomic safe harbors (GSHs) is still insufficient, making it difficult to predict the influence of gene integration on nearby genes and vice versa [143]. Similarly, genetic manipulation of *HLA* and immunosuppressive genes remain to be fully developed, validated, and likely combined with stringent safety measures prior to implementation in the clinic.

## 9. Conclusions

Cell-based therapy is a promising approach for CF and would be a less invasive alternative to transplantation, which is limited by organ shortage. Autologous cells could be harvested, gene-corrected in vitro, and transplanted back to the patient’s lungs without the need for immunosuppressive drugs. Mature cells taken from patients could be reprogrammed to iPS to facilitate their expansion and re-differentiated in vitro to proximal and distal epithelium. New technologies such as CRISPR/Cas9 have made gene editing simple and efficient where 30–50% allelic correction led to 20–50% *CFTR* function restoration in ALI culture [155]. Since CRISPR/Cas9 can have off-target effects resulting in proto-oncogene activity [156,157], the potential tumorigenicity of various gene-corrected cells could be tested in vitro prior to transplantation. For added safety, a fail-safe mechanism, such as herpes simplex virus thymidine kinase, could also be introduced to the cells which could be eliminated by ganciclovir in case of tumorigenic proliferation [149,158]. 

Many groups, including our own, have investigated ways to improve cell engraftment in the lung by various conditioning regimens to disrupt the epithelium. These treatments include naphthalene, bleomycin, polidocanol, or elastase alone or in combination with busulfan or non-lethal irradiation in animal models and have led to increased cell retention in the lung [41,159,160,161]. As we continue to have a better understanding of cell types involved in the pathogenesis of CF, we can begin to develop preconditioning regiments directly targeting specific cells within the airway epithelium. For instance, standard airway depletion with naphthalene may not be sufficient to deplete submucosal glands and will likely require a harsher insult to expose. One significant challenge that needs to be overcome is the fact that preconditioning regimens are currently in preclinical animal models and not clinically viable options. To achieve clinical translation, it will be necessary to establish clinically viable protocols to ensure adequate cell delivery and engraftment. These will likely require 3D geometric data, mapping the pulmonary vasculature and airways, and computational models that characterize fluid flow and mass transport of the human lung to guide optimization of preconditioning regimens by enabling the selection of process parameters that will minimize the existing limitations of chemical detergents. One possibility is to use ex vivo lung perfusion platform [162] to test and develop these complex therapies. 

**Figure 4:**
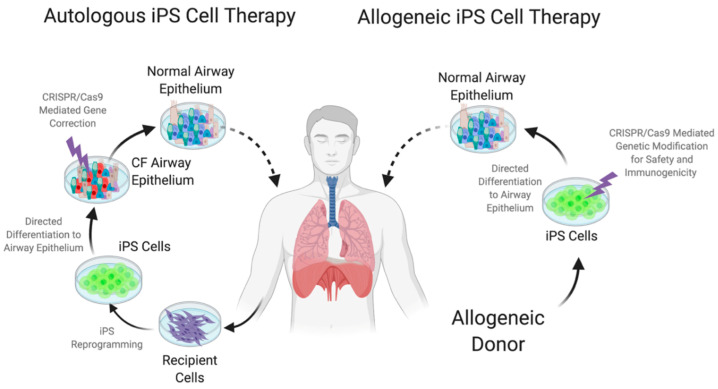
Schematic representation of cell-based therapeutic approaches using induced pluripotent stem cells. In autologous iPS cell therapy, somatic cells are isolated from the CF patient, expanded and reprogrammed to produce patient-specific induced pluripotent cells. These cells are subsequently differentiated to proximal airway epithelium, corrected for the genetic defect to achieve normal airway epithelium and transplanted back to the patient. In allogeneic iPS cell therapy, a universal iPS line (from a healthy source) which has been genetically modified for safety and immunogenicity is used to generate proximal airway epithelium which is transplanted to CF patients. Figure created with BioRender.com.

Early studies using bone marrow MSC faced controversy due to the skepticism that they could truly function as lung epithelium. With the advent of ES and iPS, which can be differentiated in vitro, viable cell sources for use in cell therapy may soon be available [65,129,130,159,163,164]. More studies will need to address the functional integration and long-term regenerative capacity of these cells as well as the safety of engrafted cells. Taken together, cell replacement therapy approaches using pluripotent cell sources in combination with genetic engineering of cells for safety and immunogenicity look to be a promising avenue for a curative treatment (Figure 4).

In conclusion, cell-based therapy for CF is a growing field and for successful future translation to the clinic, several limitations must be addressed. These include (1) development of animal models which better recapitulate the human disease, (2) clinically relevant conditioning regimens to enhance cell retention and engraftment, (3) evaluation of autologous cells, in large animal models and clinical trials (4) refinement of directed differentiation protocols to enhance the yield of proximal airway epithelial cells from pluripotent sources, and (5) assessment of feasibility in use of designer cells in CF models. Nevertheless, cell replacement therapy for CF has huge implications and may significantly increase the quality of life for patients. A strategy in which targeted removal of genetically impaired epithelial cells, followed by repopulation with a gene-corrected cohort, could result in ‘re-designed’ lungs, obviating the need to undergo transplantation.

## Abbreviations

AEC-II	Alveolar type II epithelial cells
ALI	Air–liquid-interface
BMC	Bone marrow cells
BMP4	Bone Morphogenetic Protein 4
cAMP	Cyclic adenosine monophosphate
Cas9	CRISPR associated protein 9
CCSP	Club cell secretory protein
CFBE	CF bronchial epithelial
CF	Cystic Fibrosis
*CFTR*	Cystic fibrosis transmembrane conductance regulator
Ciita	Class II, major histocompatibility complex, transactivator
Cl^−^	Chloride anion
cMyc	c-Myelocytomatosis oncogene cellular homolog
CRISPR	Clustered regularly interspaced short palindromic repeats
CTLA4-Ig	Cytotoxic T-lymphocyte-associated protein 4-immunoglobulin
ES	Embryonic stem
EVs	Extracellular vesicles
FoxI1	Forkhead box I1
GCV	Ganciclovir
GSHs	Genomic safe harbors
HLA	Human leukocyte antigens
HO-1	Heme oxygenase 1
hPSCs	Human pluripotent stem cells
HSC	Hematopoietic stem cells
HSV-TK	Herpes Simplex Virus type 1 thymidine kinase
IBMX	3-isobutyl-1-methylxanthine
IL-1β	Interleukin 1 beta
IL-10	Interleukin 10
IL-6	Interleukin 6
IL-8	Interleukin 8
IL-1ra	Interleukin-1 receptor antagonist
iPL	Induced Progenitor-Like Cells
iPS	Induced pluripotent stem
KLF4	Kruppel Like Factor 4
mRNA	Messenger Ribonucleic acid
MSC	Mesenchymal stem cells
MUC5AC	Mucin 5AC, Oligomeric Mucus/Gel-Forming
NF-κB	Nuclear factor-κB
NKX2-1	NK2 Homeobox 1
Oct-4	Octamer-binding transcription factor 4
ΔF508	Deletion of phenylalanine 508
PNEC	Pulmonary neuroendocrine cells
PPARγ	Peroxisome proliferator-activated receptor γ
Sox2	SRY (sex determining region Y)-box 2
TNFα	Tumor necrosis factor alpha
wt*CFTR*	Wild-type cystic fibrosis transmembrane conductance regulator
ZO-1	Zonula occludens-1
